# High-Temperature Tolerance of Photosynthesis Can Be Linked to Local Electrical Responses in Leaves of Pea

**DOI:** 10.3389/fphys.2017.00763

**Published:** 2017-09-29

**Authors:** Vladimir Sukhov, Vladimir Gaspirovich, Sergey Mysyagin, Vladimir Vodeneev

**Affiliations:** Department of Biophysics, N.I. Lobachevsky State University of Nizhny Novgorod, Nizhny Novgorod, Russia

**Keywords:** heating, pea seedling, plant adaptation, thermal tolerance of photosynthesis, local electrical responses

## Abstract

It is known that numerous stimuli induce electrical signals which can increase a plant's tolerance to stressors, including high temperature. However, the physiological role of local electrical responses (LERs), i.e., responses in the zone of stimulus action, in the plant's tolerance has not been sufficiently investigated. The aim of a current work is to analyze the connection between parameters of LERs with the thermal tolerance of photosynthetic processes in pea. Electrical activity and photosynthetic parameters in pea leaves were registered during transitions of air temperature in a measurement head (from 23 to 30°C, from 30 to 40°C, from 40 to 45°C, and from 45 to 23°C). This stepped heating decreased a photosynthetic assimilation of CO_2_ and induced generation of LERs in the heated leaf. Amplitudes of LERs, quantity of responses during the heating and the number of temperature transition, which induced the first generation of LERs, varied among different pea plants. Parameters of LERs were weakly connected with the photosynthetic assimilation of CO_2_ during the heating; however, a residual photosynthetic activity after a treatment by high temperatures increased with the growth of amplitudes and quantity of LERs and with lowering of the number of the heating transition, inducing the first electrical response. The effect was not connected with a photosynthetic activity before heating; similar dependences were also observed for effective and maximal quantum yields of photosystem II after heating. We believe that the observed effect can reflect a positive influence of LERs on the thermal tolerance of photosynthesis. It is possible that the process can participate in a plant's adaptation to stressors.

## Introduction

The generation of local electrical responses (LERs), which are transient depolarizations of the electrical potential on the plasma membrane, is a typical electrical response in the zone of actions of numerous factors, including changes in temperature (Pyatygin et al., 1992, 1996, 1999; Krol et al., 2003, 2004, 2006; Opritov et al., 2005), increase of light intensity (Bulychev and Vredenberg, 1995; Trebacz and Sievers, 1998; Pikulenko and Bulychev, 2005), action of chemical agents (Pyatygin et al., 1996, 1999; Volkov and Ranatunga, 2006), and application electrical current (Krol et al., 2006), etc. There are several types of LERs, including receptor potentials (depolarization responses, which have low magnitude and depend stimulus strength), voltage transients (light- and cold-induced depolarization responses, which have high magnitude, depend stimulus strength and can develop within refractory periods for action potential) and local action potentials (depolarization spikes, which have high amplitude and all-or-none characteristics) (Simons, 1981; Shimmen, 1997; Trebacz et al., 1997; Krol and Trebacz, 1999; Krol et al., 2003, 2004, 2006).

The generation of action potentials is connected with activation of Ca^2+^, K^+^, and anion channels (Krol et al., 2006; Trebacz et al., 2006; Beilby, 2007; Felle and Zimmermann, 2007) and with transitory inactivation of H^+^-ATPase in the plasma membrane (Sukhov and Vodeneev, 2009; Vodeneev et al., 2015; Sukhov, 2016). Other LERs, namely voltage transients, have K^+^ and anion-independent ionic mechanisms and are connected with calcium ions influxes from extra- and intracellular compartments (Krol et al., 2004); inactivation of H^+^-ATPase also participate in the generation of light-induced voltage transients (Krol and Trebacz, 1999). It is probable that mechanisms of receptor potentials are not universal; in particular, touch-induced responses can be connected with Ca^2+^ and Cl^−^ channels (Shimmen, 1997), and cooling-induced ones can be caused by a decrease in activity of H^+^-ATPase in the plasma membrane (Pyatygin et al., 1992; Pyatygin, 2004; Opritov et al., 2005).

Ionic mechanisms of LERs are similar to mechanisms of an important electrical signal—variation potential (VP). VP comprises two components: a long-term depolarization and “action potential-like” spikes (Vodeneev et al., 2015). The long-term depolarization is connected with activation of ligand-dependent and/or mechanosensitive Ca^2+^ channels and transient inactivation of H^+^-ATPase in the plasma membrane (Stahlberg et al., 2006; Fromm and Lautner, 2007; Gallé et al., 2015; Katicheva et al., 2015; Vodeneev et al., 2015); i.e., this component is similar to voltage transients and, possibly, receptor potentials. Moreover, amplitude of the long-term depolarization is dependent on stimulus strength or distance from the damaged zone (Vodeneev et al., 2011; Sukhov et al., 2013); these properties support similarity of voltage transients and, possibly, receptor potentials with the long-term depolarization. In contrast, “action potential-like” spikes are rather caused by activation of voltage-dependent Ca^2+^, anions and K^+^ channels (Vodeneev et al., 2011, 2015; Katicheva et al., 2014); i.e., they are similar to local action potential.

On the basis of these results we can suppose that VP has several mechanisms which are similar to those of different types of LERs; this supports the conventional hypothesis that VP is LER induced by propagation of chemical and/or hydraulic signals after local damage (Malone, 1994; Mancuso, 1999; Stahlberg et al., 2006; Fromm and Lautner, 2007; Vodeneev et al., 2015). It is known that propagating electrical signals, including VP, induce numerous physiological responses in plants (Fromm and Lautner, 2007; Gallé et al., 2015; Sukhov, 2016): changes in expression of genes (Stanković and Davies, 1996; Fisahn et al., 2004; Davies and Stankovic, 2006; Mousavi et al., 2013), production of phytohormones (Dziubinska et al., 2003; Hlavácková et al., 2006; Hlavinka et al., 2012; Mousavi et al., 2013), activation of respiration (Filek and Kościelniak, 1997; Sukhov et al., 2014a; Surova et al., 2016a), changes in transpiration and photosynthesis (Bulychev et al., 2004; Krupenina and Bulychev, 2007; Grams et al., 2009; Pavlovic et al., 2011; Sherstneva et al., 2015; Sukhov, 2016), decrease of phloem transport (Fromm, 1991; Furch et al., 2010), etc. The important result of electrical signal propagation is an increase in a plant's tolerance to stressors (Retivin et al., 1997; Sukhov et al., 2015b). In particular, electrical signals induce the increased tolerance of photosynthetic machinery and for the entire plant to cold and heat (Retivin et al., 1999; Sukhov et al., 2014b, 2015b; Surova et al., 2016b).

In contrast, the physiological role of LERs has not been sufficiently investigated. In particular, there were only few studies (Opritov et al., 1993; Pyatygin et al., 1996; Shepherd et al., 2008; Kenderešová et al., 2012) which showed the connection between LERs and plant tolerance to stressors. Pyatygin et al. (1996) showed that plant adaptation to chilling was connected with changes in parameters of cold-induced electrical responses; moreover, fast adaptation of the plant cell to chilling was observed after generation of LERs with depolarization spikes and it was absent after electrical changes without the spikes (Opritov et al., 1993). According to the results of Shepherd et al. (2008), application of a saline medium with a low concentration of calcium induced a periodic generation of LERs, which were accompanied by viability loss in *Chara* cells; however, recurring electrical responses and viability loss were absent under application of the saline medium with a high calcium concentration. Kenderešová et al. (2012) showed that a negative relationship between amplitude of Zn^2+^-induced LERs and tolerance of Arabidopsis to the high concentration of zinc was observed; however, the tolerance was investigated after several days under Zn^2+^ treatment and the connection between Zn^2+^-induced LERs and the tolerance of Arabidopsis can be caused by propagation of system electrical signals.

Plant tolerance to the chilling, saline medium and zinc can thus be connected with parameters of LERs induced by these stressors. However, the possible connection with LERs was not investigated for another important stressor, namely heating. Taking into account the positive influence of VP [i.e., LER induced by chemical and (or) hydraulic signals] on the tolerance to heating of undamaged parts of the plant (Sukhov et al., 2014b, 2015b; Surova et al., 2016b), the connection between the thermotolerance and parameters of heating-induced LERs is probable. Analysis of the connection in peas was a task of the current investigation.

## Materials and methods

### Plant material

Pea seedlings (14–21 days old) were used in this investigation. Seedlings were cultivated hydroponically (a half-strength Hoagland–Arnon medium) in a Binder KBW 240 plant growth chamber (Binder GmbH, Tuttlingen, Germany) at 24°C, with a 16/8-h (light/dark) photoperiod. Air humidity was not controlled.

### Electrical measurements

**Figure 1:**
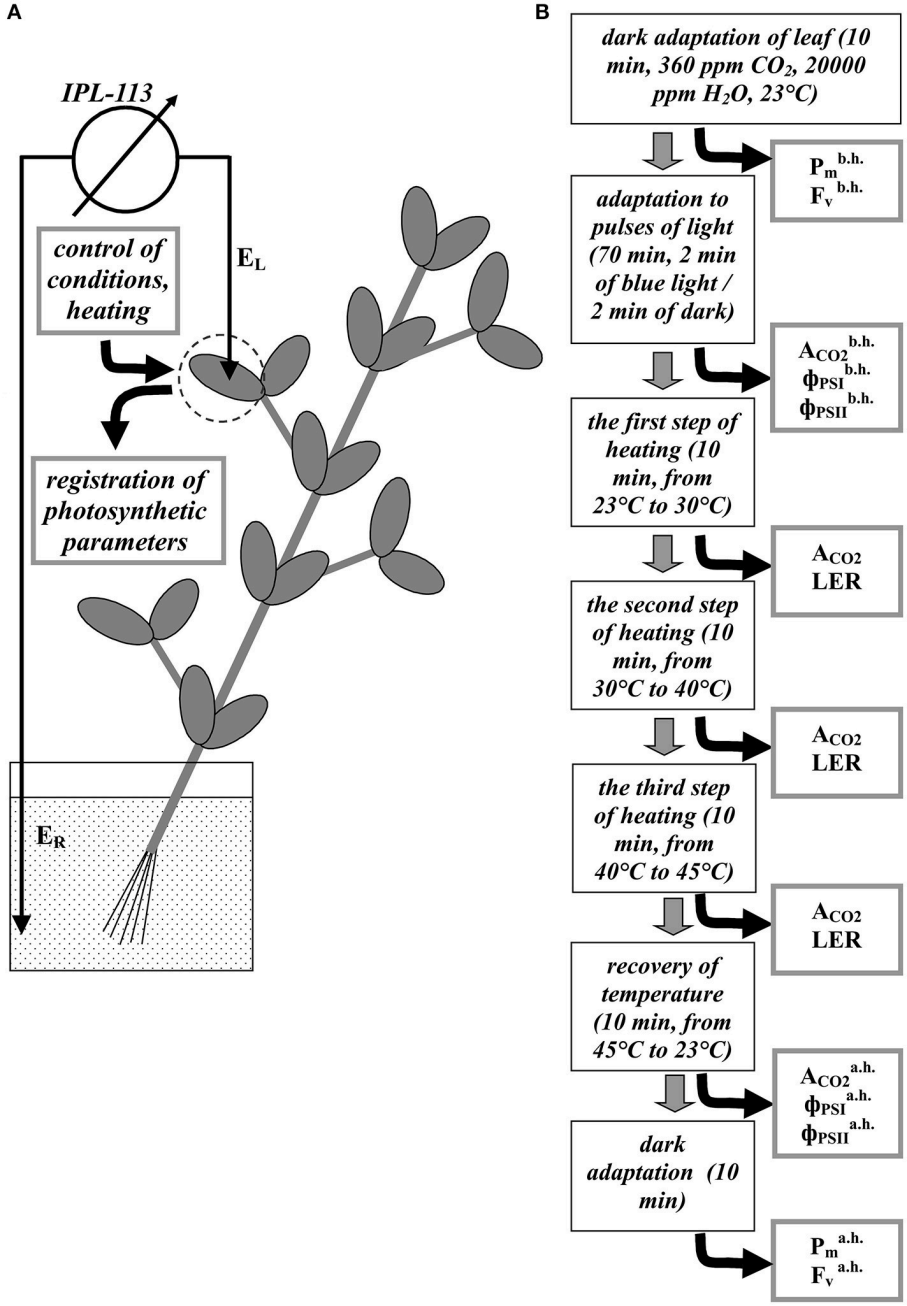
A schema of the registration of electrical and photosynthetic parameters in peas **(A)** and stages of experiment **(B)**. E_L_ is a silver electrode, E_R_ is the reference Ag^+^/AgCl electrode. P_m_, F_v_, A_CO2_, $\Phi_{\text{PSI}}^{.}$ and Φ_PSII_ are the maximal change in the P700 signal, the variable fluorescence, the photosynthetic CO_2_ assimilation rate, quantum yields of photosystem I and II, respectively. Indexes “b.h.” and “a.h” mean “before heating” and “after heating.” LER is registration of the local electrical responses.

The extracellular measurement of the electrical activity was performed using electrode consisting of a silver wire (0.5-mm diameter) and a pointed tip. The silver electrode (E_L_) was impaled into the mesophyll in between veins at the center of a leaflet (Figure 1A) in the closed photosynthesis-measuring head (see below). The reference Ag^+^/AgCl electrode (E_R_) (EVL-1M3.1, RUE “Gomel Measuring Equipment Plant,” Gomel, Belarus) was placed in a standard solution (1 mM KCl. 0.5 mM CaCl_2_, 0.1 mM NaCl) surrounding the root (about 100 ml); our previous works showed that the solution provides a stable contact between electrodes and plant (e.g., Vodeneev et al., 2011, 2017). All electrodes were connected with a high-impedance (10^12^ Ohm) amplifier IPL-113 (Semico, Novosibirsk, Russia). Results of measurements were recorded (every 1 s) in a personal computer using a standard program of IPL-113 (param2). Plant adaptation before experiment was 70 min; the standard solution was not replaced.

### Measurements of photosynthetic parameters

A portable gas exchange measuring system GFS-3000 (Heinz Walz GmbH, Effeltrich, Germany), a measuring system for the simultaneous assessment of P700 oxidation and chlorophyll fluorescence Dual-PAM-100 (Heinz Walz GmbH), and a measuring head Cuvette 3010-Dual (Heinz Walz GmbH) were used to measure photosynthetic parameters and to control conditions in the second mature leaf (Figure 1A). The CO_2_ concentration was 360 ppm; the relative humidity at 23°C was about 70%; temperature in the measuring head was varied (see below). We used pulses of actinic light (239 μmol m^−2^ s^−1^, 460 nm, 1 min); duration of dark interval between the pulses was 1 min. The durations of actinic light pulses and dark intervals were enough for stabilization of the CO_2_ assimilation rate after transitions in the light regime.

The photosynthetic parameters were measured similarly with our previous works (Sukhov et al., 2014a,b, 2015a). The dark (F_0_) and maximal (F_m_) fluorescence yields and variable fluorescence (F_V_) (Maxwell and Johnson, 2000; Kalaji et al., 2012, 2014) were measured after the dark adaptation for 10 min (Sukhov et al., 2014b). The maximal change in the P700 signal (P_m_) of PSI, reflecting maximal P700 oxidation (Klughammer and Schreiber, 2008), was measured after the preliminary illumination by far red light for 10 s.

The steady-state (F) and maximal (${\text{F}}_{\text{m}}^{\prime }$) fluorescence yields in light (Maxwell and Johnson, 2000) and steady-state (P) and maximal (${\text{P}}_{\text{m}}^{\prime }$) P700 signals in light (Klughammer and Schreiber, 2008) were measured using saturation pulses (10,000 μmol m^−2^ s^−1^, 630 nm, 300 ms). The saturation pulses were generated before the end of the each pulse of actinic light. A quantum yield of PSI (Φ_PSI_) was calculated using the equation $\phi_{PSI}=\left({P_{m}}^{\prime }-P\right)/P_{m}$ (Klughammer and Schreiber, 2008); an effective quantum yield of PSII (Φ_PSII_) was calculated using the equation $\phi_{PSII}=\left({F_{m}}^{\prime }-F\right)/{F_{m}}^{\prime }$ (Maxwell and Johnson, 2000). All parameters were programmatically calculated by software of Dual-PAM-100.

The photosynthetic CO_2_ assimilation rate (μmol CO_2_ m^−2^ s^−1^) was measured using the GFS-3000 system. Its software programmatically calculated the CO_2_ assimilation rate according to Von Caemmerer and Farquhar (1981). We calculated photosynthetic CO_2_ assimilation rate (A _CO2_) as the difference between the CO_2_ assimilation rate under light conditions and one under dark conditions for the each pulse of actinic light.

The Cuvette 3010-Dual was used for measuring of the air temperature and the leaf surface temperature in the measuring head.

### General design of experiment

A general design of experiment is shown in Figure 1B. First, leaves were adapted in dark, after which ${\text{P}}_{\text{m}}^{\text{b}.\text{h}.}$ and ${\text{F}}_{\text{v}}^{\text{b}.\text{h}.}$, which were P_m_ and F_v_ before heating (parameters of undamaged plants), were measured. Later, after 70 min of light pulses ${\text{A}}_{\text{CO}2}^{\text{b}.\text{h}.}$, $\Phi_{\text{PSI}}^{\text{b}.\text{h}.}$ and $\Phi_{\text{PSII}}^{\text{b}.\text{h}.}$, which were A_CO2_, Φ_PSI_, and Φ_PSII_ before heating (parameters of undamaged plants), were measured. Three transitions of temperature were performed after that: from 23 to 30°C, from 30 to 40°C, and from 40 to 45°C; the duration of the each transition was 10 min. Magnitudes of A_CO2_ were measured at the end of the each step of heating. After the stepped heating, temperature was recovered to 23°C (10 min); residual ${\text{A}}_{\text{CO}2}^{\text{a}.\text{h}.}$, $\Phi_{\text{PSI}}^{\text{a}.\text{h}.}$ and $\Phi_{\text{PSII}}^{\text{a}.\text{h}.}$, which were A_CO2_, Φ_PSI_, and Φ_PSII_ after heating (parameters of damaged plants), were measured after the recovery. Residual ${\text{P}}_{\text{m}}^{\text{b}.\text{h}.}$ and ${\text{F}}_{\text{v}}^{\text{b}.\text{h}.}$, which were P_m_, and F_v_ after heating (parameters of damaged plants), were measured after 10 min of the dark adaptation at 23°C. LERs were registered during the each step of the heating.

It should be noted that we preliminary investigated the residual photosynthetic CO_2_ assimilation in variants with one temperature transition (from 23 to 30°C), with two temperature transitions (from 23 to 30°C and from 30 to 40°C), and with three temperature transitions (from 23 to 30°C, from 30 to 40°C, and from 40 to 45°C). It was shown that ${\text{A}}_{\text{CO}2}^{\text{a}.\text{h}.}$ was not distinguished from ${\text{A}}_{\text{CO}2}^{\text{b}.\text{h}.}$ in variant with temperature recovery after one step of heating; decrease of this residual assimilation was not significant after two temperature transitions (the relative ${\text{A}}_{\text{CO}2}^{\text{a}.\text{h}.}$ was 88 ± 5%, *n* = 6, *p* > 0.05); significant decrease of ${\text{A}}_{\text{CO}2}^{\text{a}.\text{h}}$ was only observed after three transitions (the relative ${\text{A}}_{\text{CO}2}^{\text{a}.\text{h}}$ was 65 ± 4%, *n* = 9, *p* < 0.05). On basis of this result we concluded that only third temperature transition induced long-term damage of photosynthetic processes (suppression of photosynthetic assimilation was observed after the temperature recovery). This long-term damage is harmful for plant life and can decrease plant productivity; changes in magnitude of the damage reflect changes in plant thermotolerance. As a result variant with three steps of heating was only used in the subsequent work. The stepped heating was used as model of gradual changes in temperature which are observed under environmental conditions, because rate of the temperature increase by the measurement system can not be regulated.

The relative rate of the residual photosynthetic CO_2_ assimilation and quantum yields of photosystem I and II after heating were used for estimation of a heating-induced suppression of photosynthesis. Residual relative P_m_, F_v_ and F_v_/F_m_ were used for estimation of a heating-induced damage of photosystems I and II.

### Statistics

A separate seedling of pea was used for the each experiment with heating; the total number of plants was 40. Results of experiments were grouped according to different criteria (see Results). Quantities of repetitions in the groups are shown in the figures. Representative records, mean values, standard errors, scatter plots and correlation coefficients are presented in the figures.

## Results

### A heating-induced suppression of the photosynthetic assimilation of CO_2_ in leaves

**Figure 2:**
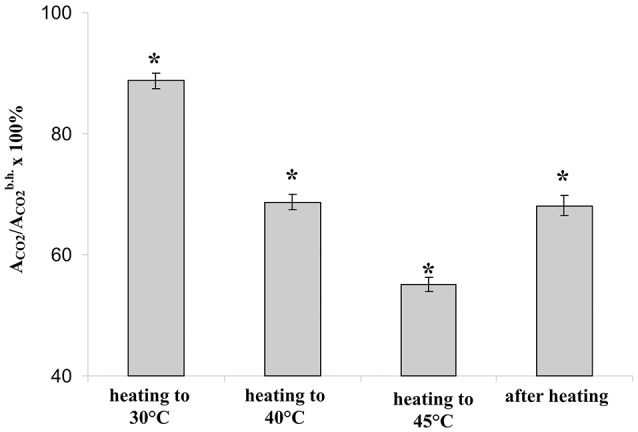
The relative photosynthetic assimilation of CO_2_ at transitions of temperature from 23 to 30°C, from 30 to 40°C, and from 40 to 45°C and after recovery of temperature from 45 to 23°C (*n* = 40). Duration of steps of heating and recovery of temperature were 10 min; A_CO2_ were measured at the end of each time interval. ^*^p < 0.05 compared with 100%, Student *t*-test.

First, we investigated suppression of the photosynthetic assimilation of CO_2_ under stepped heating and following it (Figure 2). It was shown that the temperature transition from 23 to 30°C induced a slight decrease of A_CO2_ (about 11%); the transition from 30 to 40°C caused a moderate suppression of the photosynthetic CO_2_ assimilation (about 31%); the last heating step strongly suppressed the assimilation (about 45%). A_CO2_ was increased with decrease of air temperature (about 5 min); after that it was approximately constant. After recovery of temperature (10 min) residual A_CO2_ were lower than the control value; the decrease was about 32%.

The stepped heating induced suppression of photosynthesis; moreover, the small significant decrease of photosynthetic assimilation of CO_2_ was observed even under the heating from 23 to 30°C. As a result, each temperature transition of the stepped heating decreases photosynthetic activity; the stepped heating can thus be used in a further analysis.

### Local electrical responses in leaves induced by stepped heating in peas

**Figure 3:**
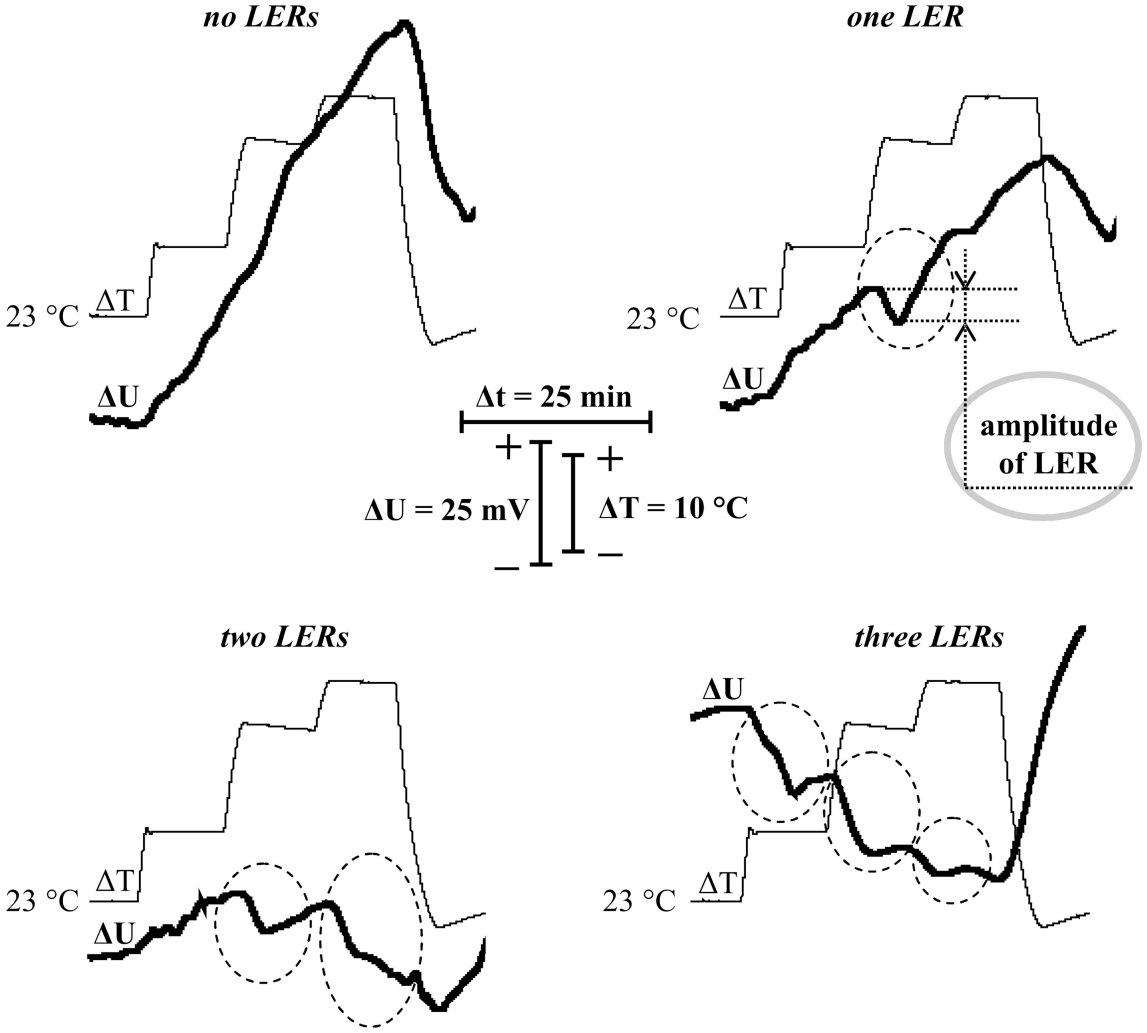
Records of surface potential changes (ΔU) under the stepped heating (ΔT) with different quantities of local electrical responses (LERs). Dotted ovals indicate LERs. A gray solid oval indicates example of measurement of the LER amplitude. Depolarization corresponds to a negative shift of the surface potential.

Analysis of the dynamics of the surface electrical potential showed that the stepped heating induced generation of LERs in most of the experiments (in 37 experiments out of 40 or 92.5%). Figure 3 showed various dynamics of the surface potential: without LERs (Figure 3A) and with one (Figure 3B), two (Figure 3C) and three (Figure 3D) LERs. LERs were transient depolarizations; its average amplitude was 10 ± 1 mV, duration in most of the variants was more than 10 min. Amplitudes of LERs, induced by different temperature transitions, were not significantly differed. High variability of LERs amplitude was similar with high variability of VP which is considered as LER induced by hydraulic and/or chemical signals (Vodeneev et al., 2015). In investigation of VP, variability of its parameters simplified analysis of connection between these parameters and parameters of physiological responses (Sukhov et al., 2012, 2014b; Surova et al., 2016a); as a result variability of LERs amplitude can simplify analysis of connection between the amplitude and pea thermotolerance.

It should be noted that the stepped heating also induced hyperpolarization (increase of the surface potential), which was the most expressive in peas without LERs or with one LER. In contrast, the effect was practically absent in peas with two or three LERs. It is probable that this hyperpolarization reflected temperature activation of H^+^-ATPase in the plasma membrane, because the maximal temperature of the leaf surface was about 40.5°C (under 45°C of air temperature in the measuring head) and optimum temperature of the H^+^-ATPase activity was 35–43°C (Briskin and Poole, 1983; Dupont and Mudd, 1985; Brauer et al., 1991).

Table 1 summarizes variants of appearance of LERs at different steps of the heating. It was shown that the first temperature transition (from 23 to 30°C) induced the first LER in many experiments (45%). The second temperature transition (from 30 to 40°C) induced the first LER in 42.5% of experiments. The third temperature transition (from 40 to 45°C) induced the first LER in 5% of experiments. These results show that LER can be generated under weak or moderate heating, which is in physiological ranges and slightly suppresses photosynthesis.

**Table 1:** Distribution of LERs induced by different temperature transitions.

	**LERs were observed under 1st temperature transition (from 23 to 30°C)**		**LERs were not observed under 1st temperature transition (from 23 to 30°C)**	
	**LERs were observed under 2nd temperature transition (from 30 to 40°C)**	**LERs were not observed under 2nd temperature transition (from 30 to 40°C)**	**LERs were observed under 2nd temperature transition (from 30 to 40°C)**	**LERs were not observed under 2nd temperature transition (from 30 to 40°C)**
LERs were observed under 3rd temperature transition (from 40 to 45°C)	10 (25%)	0 (0%)	11 (27.5%)	2 (5%)
LERs were not observed under 3rd temperature transition (from 40 to 45°C)	5 (12.5%)	3 (7.5%)	6 (15%)	3 (7.5%)

**Figure 4:**
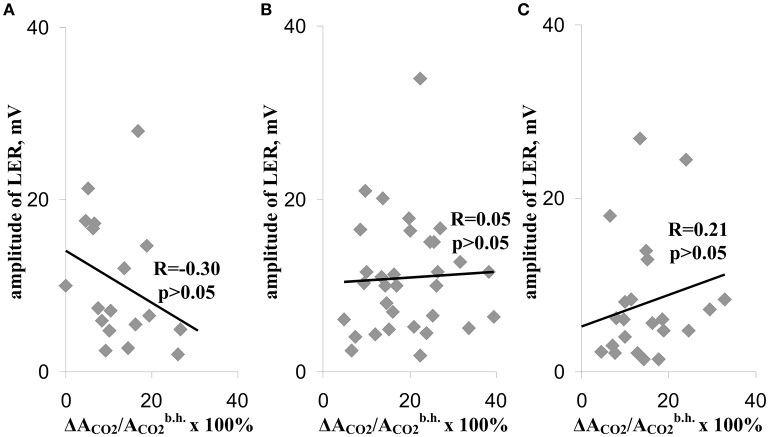
Scatter plots between amplitudes of LERs and relative decreases of the photosynthetic assimilation of CO_2_, which were observed during the different temperature transitions **(A–C)**. **(A)**, During the temperature transition from 23 to 30°C. **(B)** During the temperature transition from 30 to 40°C. **(C)** During the temperature transition from 40 to 45°C. The group with LER observed during the 1st transition included 18 peas (*n* = 18), the group with LER observed during the 2nd transition included 32 peas (*n* = 32), and the group with LER observed during the 3rd transition included 23 peas (*n* = 23). Individual amplitudes of LERs for the each temperature transition in the each plant were used. The relative decreases of the photosynthetic assimilation of CO_2_ (ΔA_CO2_/${\text{A}}_{\text{CO}2}^{\text{b}.\text{h}.}$ × 100%) were also calculated for each temperature transition in the each plant. R is Pearson's correlation coefficient.

Figure 4 shows correlations (i) between the suppression of photosynthetic CO_2_ assimilation during the first temperature transition and the amplitude of LER during this transition (Figure 4A), (ii) between the suppression of CO_2_ assimilation during the second temperature transition and the amplitude of LER during this transition (Figure 4B), and (iii) between the suppression of CO_2_ assimilation during the third temperature transition and the amplitude of LER during this transition (Figure 4C) (the method of measurement of amplitudes is shown in Figure 3). All correlations were weak, i.e., the photosynthetic damage throughout all time of the temperature increase was not connected with amplitudes of LERs. In particular, this result showed that photosynthetic suppression was not probable to induce LERs under heating.

### Analysis of connection of the residual photosynthetic CO_2_ assimilation after heating with parameters of local electrical responses in leaves

**Figure 5:**
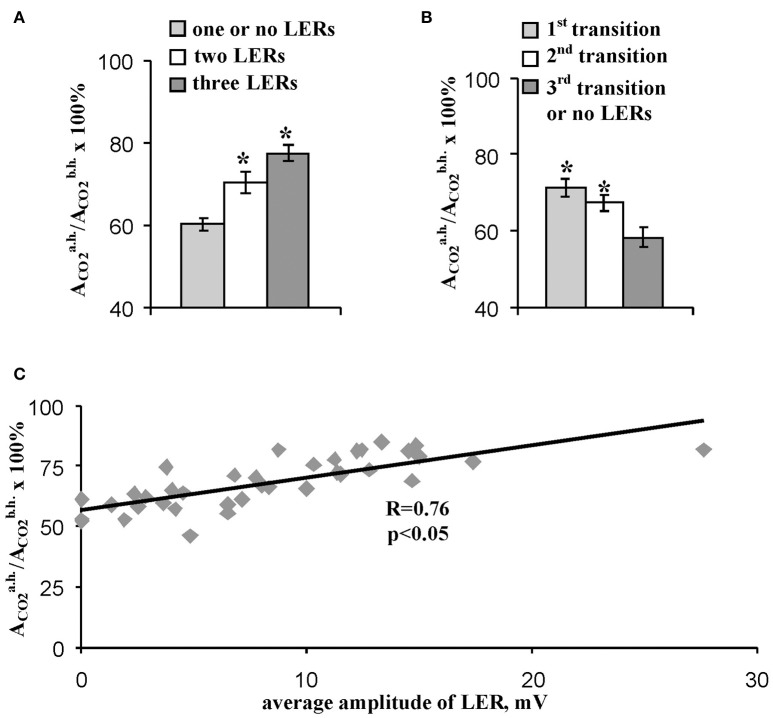
Connection of the relative residual photosynthetic assimilation of CO_2_ after heating with parameters of LERs. **(A)** The residual CO_2_ assimilation after heating in peas with different quantities of LERs during stepped heating. The group with three LERs included 10 peas (*n* = 10), the group with two LERs included 16 peas (*n* = 16), and the group with one or no LERs included 14 peas (*n* = 14). ^*^p < 0.05 compared with parameters in the group with one or no LERs, Student *t*-test. **(B)** The residual CO_2_ assimilation after heating in peas with different numbers of temperature transition, which induced the first generation of LER during the stepped heating. The group with the first LER induced by the 1st transition included 18 peas (*n* = 18), the group with the first LER induced by the 2nd transition included 16 peas (*n* = 16), and the group with the first LER induced by the 3rd transition or without LERs included 5 peas (*n* = 5). ^*^p < 0.05 compared with parameters in the group with the first LER induced by the 3rd transition or without LERs, Student *t*-test. **(C)** Scatter plots between the average amplitude of LER during stepped heating and the relative residual photosynthetic assimilation of CO_2_ after heating (*n* = 40). The average amplitude of LER was calculated for the each plant. It has been assumed that the average amplitude equaled zero when LERs were absent in the plant. R is Pearson's correlation coefficient.

The residual photosynthetic CO_2_ assimilation after temperature recovery to 23°C (in 10 min after heating) can reflect a long-term damage of photosynthesis during heating, a secondary damage of photosynthesis after heating (particularly, damage induced by increased production of reactive oxygen species) and activity of fast reparation processes of photosynthesis. Figure 5A shows that the quantity of LERs during the stepped heating was connected with the relative rate of the residual photosynthetic CO_2_ assimilation after heating: the small rate (60%) was in the group without LERs or with one response, the moderate rate (70%) was in the group with two LERs, and the high rate (78%) was in the group with three LERs. Analysis of connection of the heating-induced suppression of the photosynthetic CO_2_ assimilation with the number of the temperature transition, which induced the first generation of LER (the number was qualitatively connected with the heating threshold for generation of the electrical response) showed a similar result (Figure 5B): the relative rate of the residual photosynthetic CO_2_ assimilation after heating was maximal (71%) in the group with generation of the first LER at the first temperature transition, and it was minimal (58%) in the group with generation of the first LER at the third temperature transition or without generation of the electrical response. Figure 5C shows that the average amplitude of LER was significantly correlated with the relative rate of the residual photosynthetic CO_2_ assimilation after heating. It should be noted that plants without LERs were not separately analyzed because their quantity was small (*n* = 3); however, the average residual photosynthetic assimilation of CO_2_ after heating was 55 ± 3% in these plants, and it was lower than residual assimilation in plants with LERs (Figure 5).

These results show that residual photosynthetic activity after heating in pea leaves was strongly connected with parameters of heating-induced LERs. In consideration of absence of correlation between amplitudes of LERs and photosynthetic CO_2_ assimilation during the heating (Figure 4), parameters of LERs were strongly connected with the plant tolerance to the secondary damage of photosynthesis after heating and/or the activity of fast reparation processes of photosynthesis. Both the decreased secondary photosynthetic damage and the increased fast reparation of photosynthesis promote increased total photosynthetic thermotolerance in plant. However, the connection can be potentially caused by distinct physiological states of peas in the investigated group. As a result, in next section of work we analyzed the connection of parameters of LERs and the residual photosynthetic activity after heating in leaves with the initial rate of the photosynthetic assimilation of CO_2_, i.e., with CO_2_ assimilation before heating.

### Analysis of the connection of local electrical response parameters and photosynthesis thermotolerance with the initial rate of CO_2_ assimilation in leaves

**Figure 6:**
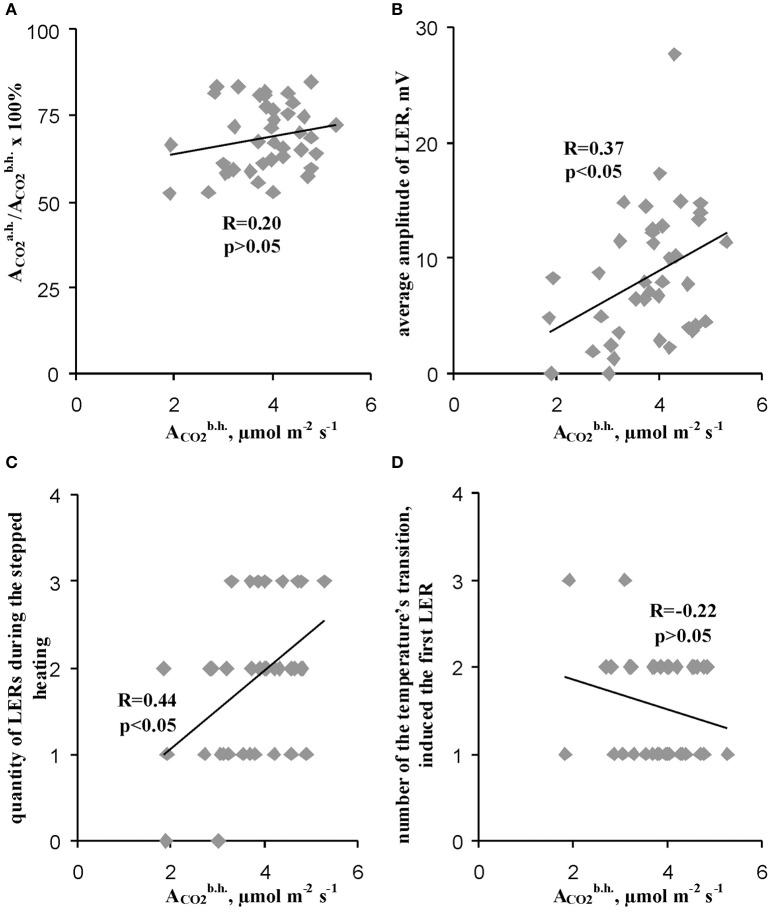
Connections of the relative residual photosynthetic assimilation of CO_2_ after heating **(A)** and parameters of LERs **(B–D)** with the rate of photosynthetic assimilation of CO_2_ before heating. **(A)** Scatter plots between the relative residual photosynthetic assimilation of CO_2_ after heating and the rate of photosynthetic assimilation of CO_2_ before heating (*n* = 40). **(B)** Scatter plots between the average amplitude of LER during the stepped heating and the rate of photosynthetic assimilation of CO_2_ before heating (*n* = 40). **(C)** Scatter plots between the quantity of LERs during the stepped heating and the rate of photosynthetic assimilation of CO_2_ before heating (*n* = 40). **(D)** Scatter plots between the number of temperature transition, which induced the first generation of LER during the stepped heating, and the rate of the photosynthetic assimilation of CO_2_ before heating (*n* = 37, experiments without LERs were not included). The average amplitude of LER was calculated for the each plant during the stepped heating. It has been assumed that the average amplitude equaled zero when LERs were absent in the plant. R is Pearson's correlation coefficient.

We used the initial rate of the photosynthetic CO_2_ assimilation in leaves (the assimilation before heating) as parameter which reflected the initial state of photosynthesis in different peas. Figure 6A shows that value of the initial rate of the photosynthetic CO_2_ assimilation in leaves was not connected with the residual relative A_CO2_ after heating (correlation was weak and was not significant), i.e., the thermotolerance of photosynthesis was not dependent on this initial rate of assimilation before heating. It should be additionally noted that Figure 6A shows that non-linear dependences (even, dependences with several extremes) between initial photosynthetic CO_2_ assimilation and the relative residual assimilation are not probable too.

In contrast, the average amplitude (Figure 6B) and the quantity of LERs during the stepped heating (Figure 6C) were significantly correlated with the initial rate of the photosynthetic CO_2_ assimilation. However, the correlation coefficients (0.37 and 0.44) were low; they were less than the coefficient of correlation between the average LER amplitude and the relative A_CO2_ after heating (0.76). The connection of the initial rate of the photosynthetic CO_2_ assimilation with the number of the temperature transition, which induced the first LER, was not significant (Figure 6D). Thus, the photosynthesis thermotolerance in leaves was not connected with the initial photosynthetic activity; the connection of parameters of LERs with this activity was weak.

### Analysis of the connection of residual parameters of photosynthetic light reactions after heating with parameters of local electrical responses in leaves

**Figure 7:**
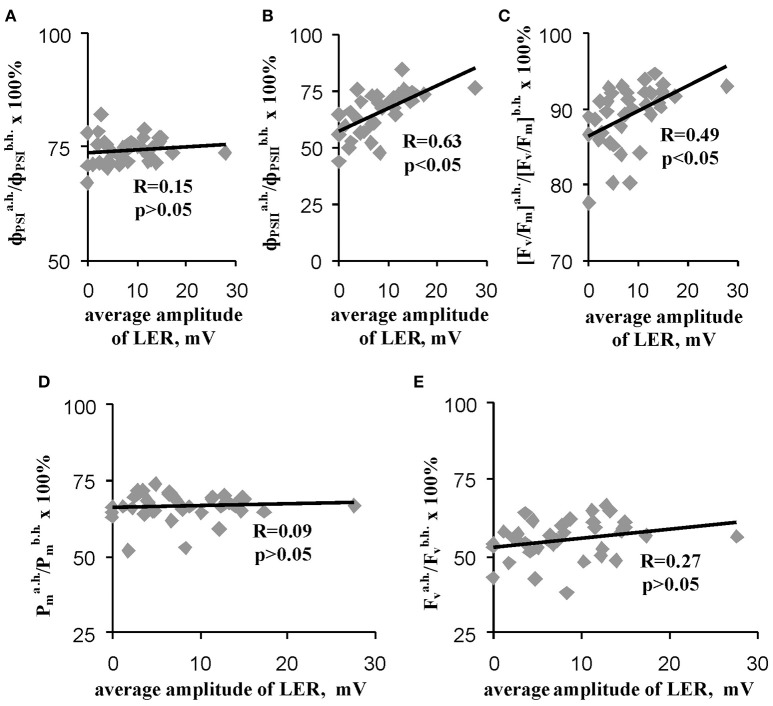
Scatter plots between average amplitudes of LER and relative quantum yields of photosystems I **(A)** and II **(B)**, the relative maximal quantum yield of photosystem II **(C)** and relative quantities of undamaged photosystems I **(D)** and II **(E)** after heating (*n* = 40). The average amplitude of LER was calculated for the each plant during the stepped heating. It has been assumed that the average amplitude equaled zero when LERs were absent in the plant. R is Pearson's correlation coefficient.

**Figure 8:**
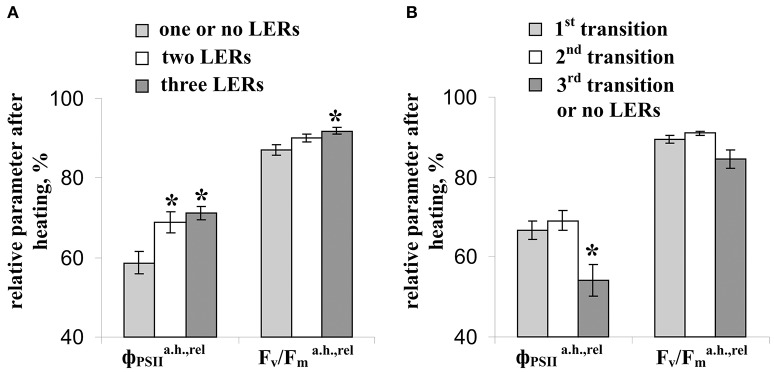
Connection of the relative effective ($\Phi_{\text{PSII}}^{\text{a}.\text{h}.,\text{rel}}$) and maximal (F_v_/${\text{F}}_{\text{m}}^{\text{a}.\text{h}.,\text{rel}}$) quantum yields of photosystem II after heating with parameters of LERs. **(A)** The relative residual effective and maximal quantum yields of photosystem II after heating in peas with different quantities of LERs during the stepped heating. The group with three LERs included 10 peas (*n* = 10), the group with two LERs included 16 peas (*n* = 16), and the group with one or no LERs included 14 peas (*n* = 14). ^*^p < 0.05 compared with parameters in the group with one or no LERs, Student *t*-test. **(B)** The relative residual effective and maximal quantum yields of photosystem II after heating in peas with a different number of temperature transition, which induced the first generation of LER during the stepped heating. The group with the first LER induced by the 1st transition included 18 peas (*n* = 18), the group with the first LER induced by the 2nd transition included 16 peas (*n* = 16), and the group with the first LER induced by the 3rd transition or without LERs included 5 peas (*n* = 5). ^*^p < 0.05 compared with parameters in the group with the first LER induced by the 3rd transition or without LERs, Student *t*-test.

Figure 7 shows that relative value of the residual effective ($\Phi_{\text{PSI}}^{\text{a}.\text{h}.}$/$\Phi_{\text{PSI}}^{\text{b}.\text{h}}$) and maximal ([F_v_/F_m_]^a.h.^/[F_v_/F_m_]^b.h.^) quantum yields of photosystem II after heating were significantly correlated with the average amplitude of LER (0.63 and 0.49). However, the correlations were moderate; i.e., the linear connection between these parameters was not very expressive. Other investigated parameters of photosynthetic light reactions, including $\Phi_{\text{PSI}}^{\text{a}.\text{h}.}$/$\Phi_{\text{PSI}}^{\text{b}.\text{h}.}$, ${\text{P}}_{\text{m}}^{\text{a}.\text{h}.}$/${\text{P}}_{\text{m}}^{\text{b}.\text{h}}$, and ${\text{F}}_{\text{v}}^{\text{a}.\text{h}.}$/${\text{F}}_{\text{v}}^{\text{b}.\text{h}.}$, were not connected with the average amplitude of LER. Figure 8 supports connection of the relative effective ($\Phi_{\text{PSI}}^{\text{a}.\text{h}.}$/$\Phi_{\text{PSI}}^{\text{b}.\text{h}}$) and maximal ([F_v_/F_m_]^a.h.^/[F_v_/F_m_]^b.h.^) quantum yields of photosystem II after heating with parameters of LERs: an increase of these parameters was connected with an increase in the quantity of LERs during the stepped heating (Figure 8A) and with a decrease in the number of the temperature transition, which induced the first generation of LER (Figure 8B). The result shows that thermotolerance of photosystem II (probably, the photosystem II tolerance to the secondary damage after heating and/or the activity of fast reparation processes in this photosystem) was connected with parameters of LERs in leaves.

### Analysis of the connection of residual photosynthetic parameters after heating with amplitudes of local electrical responses induced by different temperature transitions

**Figure 9:**
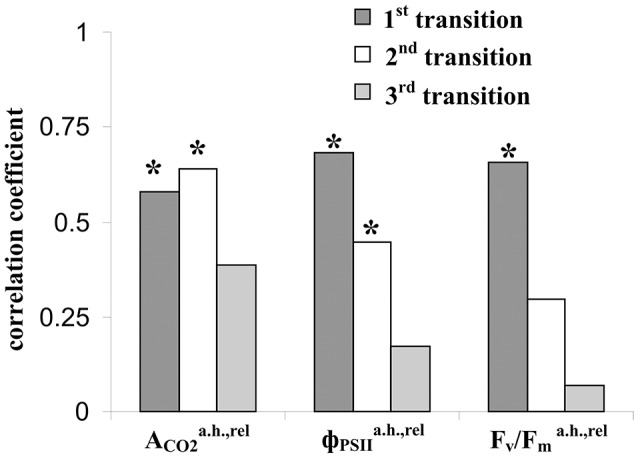
Coefficients of correlation of amplitudes of LERs, induced by different temperature transitions, with the relative residual photosynthetic assimilation of CO_2_ (${\text{A}}_{\text{CO}2}^{\text{a}.\text{h}.,\text{rel}}$), effective ($\Phi_{\text{PSII}}^{\text{a}.\text{h}.,\text{rel}}$) and maximal (F_v_/${\text{F}}_{\text{m}}^{\text{a}.\text{h}.,\text{rel}}$) quantum yields of photosystem II after heating. The group with LER observed during the 1st transition included 18 peas (*n* = 18), the group with LER observed during the 2nd transition included 32 peas (*n* = 32), and the group with LER observed during the 3rd transition included 23 peas (*n* = 23). Individual amplitudes of LERs for the each temperature transition in the each plant were used. ^*^The correlation coefficient is significant.

Figure 9 shows that the relative residual photosynthetic CO_2_ assimilation after heating was strongly correlated with the amplitudes of LERs induced by the first and second steps of heating; however, the correlation was absent for LERs induced by the third temperature transition. Residual parameters of photosystem II after heating were maximally correlated with the amplitudes of LERs induced by the first step of heating; these correlations were weak for LERs induced by the second temperature transition; it was absent for LERs induced by the third one. The result shows that connection between LERs and photosynthetic thermotolerance (the tolerance to the secondary damage after heating and/or the activity of fast reparation processes of photosynthesis) was stronger for early electrical responses.

## Discussion

A number of studies (Retivin et al., 1997, 1999; Sukhov et al., 2014b, 2015b; Surova et al., 2016b) show a positive influence of electrical signals, including VP, on plant tolerance to negative changes in temperature. Considering the similarity between mechanisms of LERs and mechanisms of the generation of VP (Vodeneev et al., 2015, 2016), it can not be excluded that LERs can also influence the tolerance of plants to heating and chilling. The connection of the generation of LERs under chilling with tolerance of plants to low temperatures was shown in some studies (Opritov et al., 1993, 1994); however, the connection of parameters of heating-induced LERs with the thermotolerance of plants was not investigated.

There are two important questions in this problem. First, we previously showed that VP increased the thermotolerance of photosystem I and the whole plant (Sukhov et al., 2014b, 2015b; Surova et al., 2016b). However, VP was caused by the damaging thermal stimulus (burn), which is a standard stimulus for the induction of this electrical signal (Hlavácková et al., 2006; Grams et al., 2009; Katicheva et al., 2014; Vodeneev et al., 2015). Thus, the first important question is “Can electrical responses be generated under physiological heating?” Our previous results (Vodeneev et al., 2017) showed that heating to 52°C induced propagation of VP. Results of the current work showed that even the slight heating to 30°C (the temperature of the leaf surface was about 28.9°C) induced generation of electrical responses in the heated zone in 45% of peas; after second heating to 40°C (temperature of leaf surface was about 37.1°C), the first LER were observed in 42.5% of peas (Figure 3, Table 1). This result means that heating-induced LERs can be induced under slightly increased temperatures. It is unlikely that these LERs were local action potentials or voltage transients; because action potential and voltage transients have high amplitudes, additionally, action potential has an all-or-none characteristic (Krol and Trebacz, 1999). The revealed LERs were rather similar with receptor potentials with low amplitudes (Krol and Trebacz, 1999) and different mechanisms of generation (Pyatygin et al., 1992; Shimmen, 1997; Pyatygin, 2004; Opritov et al., 2005).

The mechanism of revealed LERs requires additional investigation; it is unlikely that it is connected with temperature-dependent damage of H^+^-ATPase in the plasma membrane because temperature optimum of the enzyme is 35–43°C (Briskin and Poole, 1983; Dupont and Mudd, 1985; Brauer et al., 1991). The decrease in photosynthetic CO_2_ assimilation, which is observed in our experiments (Figure 2), is also unlike the mechanism of generation of LERs, because their amplitudes are not correlated with magnitudes of this decrease (Figure 4). On the other hand, the influence of increased temperature can be connected with changes in the plasma membrane conductivity (Brauer et al., 1991). In particular, Saidi et al. (2009) showed that a temperature increase to 28 and 32°C activates Ca^2+^ channels in moss plants; moreover, a temperature increase to 38°C induces transient increase of cytoplasmic Ca^2+^ concentration in the range of minutes after initiation of heating, and the response continues for at least 20 min. This long-term increase of Ca^2+^ concentration is in a good accordance with simulated dynamics of calcium ions during VP, which causes inactivation of H^+^-ATPase in the plasma membrane (Sukhov et al., 2013; Sukhova et al., 2017), and with stimuli-induced propagating Ca^2+^ waves (Choi et al., 2014; Kiep et al., 2015), which is possible to be also connected with generation of VP. Thus, we can speculate that LERs, induced by the stepped heating, can be connected with Ca^2+^ influx and a decrease in H^+^-ATPase activity.

The second question is “Is heating LERs connected with thermotolerance of plants?” Our results showed that parameters of heating-induced LERs (amplitudes, quantity of electrical responses, temperature threshold) were significantly connected with the residual photosynthetic CO_2_ assimilation (Figures 5, 9) and effective and maximal quantum yields of photosystem II (Figures 7–9) after heating; i.e., the tolerance of dark reactions of photosynthesis and light reactions in photosystem II to the increased temperature was linked to electrical responses. Probably, this increased tolerance can be connected with decrease of the secondary damage of photosynthetic processes and/or with activation of the fast photosynthetic reparation after heating because the photosynthetic CO_2_ assimilation during the heating, which reflected primary photosynthetic suppression, was not connected with parameters of LERs. The supposition is in a good accordance with literature data; in particular, electrical signals stimulated photosynthetic reparation processes (Retivin et al., 1999; Surova et al., 2016b) and LERs induced recovery of the membrane potential (Opritov et al., 1993). In contrast, the thermotolerance of photosystem I was not connected with the parameters of LERs.

The connection between parameters of LERs and photosynthetic thermotolerance can be explained using three hypotheses: (i) Increase of the photosynthetic damage suppresses the generation of LERs; (ii) Generation of LERs decreases the photosynthetic damage; and (iii) Stimulation of generation of LERs and decrease of the photosynthetic damage do not interact, but they reflect a third unknown process. The first hypothesis is unlikely because correlation between suppression of photosynthesis and parameters of LERs was absent during any temperature transitions (Figure 4). Moreover, inactivation of photosynthesis can rather stimulate depolarization (decrease of photosynthetic activity induces depolarization of the plasma membrane potential e.g., Miedema and Prins, 1993). Thus, the connection between residual photosynthetic parameters after heating and parameters of LERs can not be caused by photosynthetic suppression during heating.

Our results definitely do not support the second or third hypothesis; however, there are some points of benefit of the second one. Firstly, the first LER was often generated during the first temperature transition from 23 to 30°C (45% of experiments, Table 1); i.e., their generation preceded the main thermal damage of photosynthesis (temperature transitions from 30 to 40°C and, especially, 40 to 45°C, Figure 2). Additionally, amplitudes of this “early” LERs induced by the first temperature transitions were significantly correlated (0.58–0.68) with the residual photosynthetic CO_2_ assimilation and quantum yields of photosystem II after heating (Figure 9).

Second, the connection of the amplitudes of LERs induced by different temperature transitions with residual photosynthetic parameters after heating was decreased with the increase of the transition number (Figure 9); in particular, LERs induced by the third temperature transition (which mainly suppressed photosynthesis, Figure 2) were not connected with residual photosynthetic activity after heating. The hypothesis about the positive influence of LERs on the photosynthetic thermotolerance can explain the result in case of development for 10–20 min of LER-induced increase of the thermotolerance. Two possible chains of events can occur: (i) LERs induced by the first or the second temperature transition → development of the increased photosynthetic thermotolerance (10–20 min) → the weak damage of photosynthesis under the third transition and the active photosynthetic reparation after heating, or (ii) LERs induced by the third temperature transition → the low photosynthetic thermotolerance (it had not time to increase) and strong damage of photosynthesis under the third transition. In this connection it should be also noted that a positive effect of electrical signals on plant tolerance to gradual cooling was developed in 15–25 min after stimulation (Retivin et al., 1997); the increased of photosynthetic thermotolerance was observed in 15 min after induction of VP (Sukhov et al., 2014b, 2015b) and action potential (Retivin et al., 1999). The alternative hypothesis requires several independent mechanisms of temperature influence on parameters of LERs and photosynthetic thermotolerance. These mechanisms should explain (i) the similar influence of temperature on the residual photosynthetic CO_2_ assimilation after heating and LERs, induced by transitions from 23 to 30°C or from 30 to 40°C, and absence of this similarity for LERs, induced by transition from 40 to 45°C, and (ii) the similar influence of temperature on residual quantum yields of photosystem II after heating and LERs, induced by transition from 23 to 30°C, and absence of this similarity for LERs, induced by transitions from 30 to 40°C or from 40 to 45°C. We can not exclude these possible mechanisms; however, the hypothesis about influence of LERs on the photosynthetic thermotolerance seems to be simpler.

Third, Figures 6B,C show that the initial rate of photosynthetic CO_2_ assimilation was significantly connected with parameters of LERs. If the initial state of a pea independently influenced LERs parameters and photosynthetic thermotolerance, then it can be expected that the initial A_CO2_ would be significantly connected with the thermotolerance. However, the initial A_CO2_ before heating was not correlated with the relative residual photosynthetic CO_2_ assimilation after heating (Figure 6A).

Additionally, many potential mechanisms of LER generation can be connected with mechanisms of tolerance to stressors, including photosynthetic tolerance; in particular, the Ca^2+^ and H^+^ influxes can stimulate different mechanisms of protection of photosynthetic machinery (Müller et al., 2001; Hochmal et al., 2015; Sukhov, 2016), and the K^+^ efflux can participate in protection of the plasma membrane (Opritov et al., 1994). In particular, the indirect argument, which supports the hypothesis of LER influence on photosynthetic thermotolerance, is a modification of tolerance of photosynthetic processes induced by other electrical responses—propagating electrical signals (Sukhov, 2016). It is known that action potential can increase the tolerance of photosystem II to heating and cooling (Retivin et al., 1999), and VP increases thermotolerance of photosystem I (Sukhov et al., 2014b, 2015b; Surova et al., 2016b). These changes in photosynthetic thermotolerance are connected with photosynthetic responses, induced by electrical signals (Sukhov et al., 2014b; Sukhov, 2016). In particular, an increase in thermotolerance can be caused by electrical signal-induced stimulation of the cyclic electron flow (Sukhov et al., 2015a) and the non-photochemical quenching in photosystem II (Krupenina and Bulychev, 2007; Pavlovic et al., 2011; Sukhov et al., 2012, 2014a, 2016). Both processes decrease production of reactive oxygen species and, thereby, increase tolerance to secondary photosynthetic damage (Roach and Krieger-Liszkay, 2014; Sukhov, 2016). Also, electrical signals induce an increase in ATP content in leaves and stem (Sukhov, 2016; Surova et al., 2016a), which can participate in reparation of photosystem II after damage (Allakhverdiev et al., 2005a,b, 2008). Influence of electrical signals on photosynthetic thermotolerance can be also connected with other physiological processes including changes in a stomata opening (Sukhov et al., 2015b), which can be regulated by electrical signals (Grams et al., 2009; Sukhov et al., 2012, 2015b). It should be additionally noted that time of development of VP-induced changes in cyclic electron flow, the non-photochemical quenching in photosystem II, the ATP content and the stomata opening was about 5–10 min after propagation of VP in peas (Sukhov et al., 2014b, 2015a; Surova et al., 2016a).

There are two potential mechanisms of initiation of photosynthetic responses in plants (Sukhov, 2016), including electrical signals-induced increase of the cyclic electron flow and the non-photochemical quenching. The first is Ca^2+^ influx, which participates in the generation of action potential and VP (Sukhov and Vodeneev, 2009; Vodeneev et al., 2015), and increases the concentration of calcium ions in cytoplasm. According to a hypothesis of Krupenina and Bulychev (2007) Ca^2+^ transports to the stroma of chloroplasts and inactivates Calvin-Benson cycle enzymes. An alternative hypothesis considers the influx of H^+^ and changes in intra- and extracellular pH as the main mechanism of induction of photosynthetic responses (Sukhov, 2016). There are several potential ways that proton signals influence photosynthesis: (i) increased apoplastic pH can decrease CO_2_ flow to mesophyll cells and suppress photosynthetic dark reactions (Sherstneva et al., 2016a,b), which induces inactivation of light reactions (Pavlovic et al., 2011; Sukhov et al., 2012, 2014a); (ii) decreased pH in cytoplasm (Sukhov et al., 2014a) contributes to decreased pH in stroma and the lumen of chloroplasts (Sukhov et al., 2016), which affects photosynthetic light reactions, decreasing linear electron flow, increasing the non-photochemical quenching, and, probably, changing the location of the ferredoxin-NADP-reductase (Sukhov, 2016).

Thus, fluxes of Ca^2+^ and H^+^, which are key participants in different types of LERs, including receptor potentials (Pyatygin et al., 1992; Shimmen, 1997; Pyatygin, 2004; Opritov et al., 2005), voltage transients (Krol and Trebacz, 1999; Krol et al., 2004), and local action potentials (Krol et al., 2006; Trebacz et al., 2006; Beilby, 2007; Felle and Zimmermann, 2007), can induce responses of photosynthesis, which increases tolerance of photosynthetic machinery to increased temperatures (Sukhov et al., 2014b) and, possibly, stimulates its reparation (Surova et al., 2016a,b). Taken together, these results show that the positive influence of LERs on photosynthetic thermotolerance (the tolerance to the secondary damage after heating and/or the activity of fast reparation processes of photosynthesis) is a probably explanation of the connection between these electrical responses and tolerance to increased temperatures, which was shown in current work. However, the hypothesis requires further investigation, particularly, with using inhibitor analysis, methods of molecular biology, or genetical tools.

## Author contributions

VS: Design of experiment, analysis of data, preparation of manuscript; VG: Performance of experiments; SM: Participation in analysis of data; VV: Participation in analysis of data, participation in preparation of manuscript.

### Conflict of interest statement

The authors declare that the research was conducted in the absence of any commercial or financial relationships that could be construed as a potential conflict of interest.

## References

[B1] Allakhverdiev S. I. Nishiyama Y. Takahashi S. Miyairi S. Suzuki I. Murata N. (2005a). Systematic analysis of the relation of electron transport and ATP synthesis to the photodamage and repair of photosystem II in *Synechocystis*. Plant Physiol. 137, 263–273. 10.1104/pp.104.054478 15618415 PMC548857

[B2] Allakhverdiev S. I. Tsvetkova N. Mohanty P. Szalontai B. Moon B. Y. Debreczeny M.. (2005b). Irreversible photoinhibition of photosystem II is caused by exposure of *Synechocystis* cells to strong light for a prolonged period. Biochim. Biophys. Act. 1708, 342–351. 10.1016/j.bbabio.2005.05.006 15950925

[B3] Allakhverdiev S. I. Kreslavski V. D. Klimov V. V. Los D. A. Carpentier R. Mohanty P. (2008). Heat stress: an overview of molecular responses in photosynthesis. Photosyn. Res. 98, 541–550. 10.1007/s11120-008-9331-0 18649006

[B4] Beilby M. J. (2007). Action potential in Charophytes. Int. Rev. Cytol. 257, 43–82. 10.1016/S0074-7696(07)57002-6 17280895

[B5] Brauer D. Loper M. Schubert C. Tu S. I. (1991). Effects of temperature on the coupled activities of the vanadate-sensitive proton pump from maize root microsomes. Plant Physiol. 96, 1114–1117. 10.1104/pp.96.4.1114 16668306 PMC1080901

[B6] Briskin D. P. Poole R. J. (1983). Characterization of a K^+^-stimulated adenosine triphosphatase associated with the plasma membrane of red beet. Plant Physiol. 71, 350–355. 10.1104/pp.71.2.350 16662829 PMC1066036

[B7] Bulychev A. A. Kamzolkina N. A. Luengviriya J. Rubin A. B. Müller S. C. (2004). Effect of a single excitation stimulus on photosynthetic activity and light-dependent pH banding in Chara cells. J. Membr. Biol. 202, 11–19. 10.1007/s00232-004-0716-5 15702376

[B8] Bulychev A. A. Vredenberg W. J. (1995). Enchancement of the light-triggered electrical response in plant cells following their de-enegisation witch uncouplers. Physiol. Plant. 94, 64–70. 10.1111/j.1399-3054.1995.tb00785.x

[B9] Choi W. G. Toyota M. Kim S. H. Hilleary R. Gilroy S. (2014). Salt stress-induced Ca^2+^ waves are associated with rapid, long-distance root-to-shoot signaling in plants. Proc. Natl. Acad. Sci. U.S.A. 111, 6497–6502. 10.1073/pnas.1319955111 24706854 PMC4035928

[B10] Davies E. Stankovic B. (2006). Electrical signals, the cytoskeleton, and gene expression: a hypothesis on the coherence of the cellular responses to environmental insult, in Communication in Plants. Neuronal Aspects of Plant Life, eds Baluška F. Mancuso S. Volkmann D. (Berlin; Heidelberg; New York, NY: Springer-Verlag), 309–320.

[B11] Dupont F. M. Mudd J. B. (1985). Acclimation to low temperature by microsomal membranes from tomato cell cultures. Plant Physiol. 77, 74–78. 10.1104/pp.77.1.74 16664031 PMC1064459

[B12] Dziubinska H. Filek M. Koscielniak J. Trebacz K. (2003). Variation and action potentials evoked by thermal stimuli accompany enhancement of ethylene emission in distant non-stimulated leaves of *Vicia faba* minor seedlings. J. Plant Physiol. 160, 1203–1210. 10.1078/0176-1617-00914 14610889

[B13] Felle H. H. Zimmermann M. R. (2007). Systemic signaling in barley through action potentials. Planta 226, 203–214. 10.1007/s00425-006-0458-y 17226028

[B14] Filek M. Kościelniak J. (1997). The effect of wounding the roots by high temperature on the respiration rate of the shoot and propagation of electric signal in horse bean seedlings (*Vicia faba* L. minor). Plant Sci. 123, 39–46. 10.1016/S0168-9452(96)04567-0

[B15] Fisahn J. Herde O. Willmitzer L. Peña-Cortés H. (2004). Analysis of the transient increase in cytosolic Ca^2+^ during the action potential of higher plants with high temporal resolution: requirement of Ca^2+^ transients for induction of jasmonic acid biosynthesis and PINII gene expression. Plant Cell Physiol. 45, 456–459. 10.1093/pcp/pch054 15111720

[B16] Fromm J. (1991). Control of phloem unloading by action potentials in Mimosa. Physiol. Plant. 83, 529–533. 10.1111/j.1399-3054.1991.tb00130.x

[B17] Fromm J. Lautner S. (2007). Electrical signals and their physiological significance in plants. Plant Cell Environ. 30, 249–257. 10.1111/j.1365-3040.2006.01614.x 17263772

[B18] Furch A. C. Zimmermann M. R. Will T. Hafke J. B. van Bel A. J. (2010). Remote-controlled stop of phloem mass flow by biphasic occlusion in *Cucurbita maxima*. J. Exp. Bot. 61, 3697–3708. 10.1093/jxb/erq181 20584788 PMC2921205

[B19] Gallé A. Lautner S. Flexas J. Fromm J. (2015). Environmental stimuli and physiological responses: the current view on electrical signalling. Environ. Exp. Bot. 114, 15–21. 10.1016/j.envexpbot.2014.06.013

[B20] Grams T. E. E. Lautner S. Felle H. H. Matyssek R. Fromm J. (2009). Heat-induced electrical signals affect cytoplasmic and apoplastic pH as well as photosynthesis during propagation through the maize leaf. Plant Cell Environ. 32, 319–326. 10.1111/j.1365-3040.2008.01922.x 19054346

[B21] Hlavácková V. Krchnák P. Nauš J. Novák O. Špundová M. Strnad M. (2006). Electrical and chemical signals involved in short-term systemic photosynthetic responses of tobacco plants to local burning. Planta 225, 235–244. 10.1007/s00425-006-0325-x 16773374

[B22] Hlavinka J. NoŽková-Hlavácková V. Floková K. Novák O. Nauš J. (2012). Jasmonic acid accumulation and systemic photosynthetic and electrical changes in locally burned wild type tomato, ABA-deficient sitiens mutants and sitiens pre-treated by ABA. Plant Physiol. Biochem. 54, 89–96. 10.1016/j.plaphy.2012.02.014 22391126

[B23] Hochmal A. K. Schulze S. Trompelt K. Hippler M. (2015). Calcium-dependent regulation of photosynthesis. Biochim. Biophys. Acta 1847, 993–1003. 10.1016/j.bbabio.2015.02.010 25687895

[B24] Kalaji H. M. Goltsev V. Bosa K. Allakhverdiev S. I. Strasser R. J. Govindjee. (2012). Experimental *in vivo* measurements of light emission in plants: a perspective dedicated to David Walker. Photosyn. Res. 114, 69–96. 10.1007/s11120-012-9780-3 23065335

[B25] Kalaji H. M. Schansker G. Ladle R. J. Goltsev V. Bosa K. Allakhverdiev S. I.. (2014). Frequently asked questions about *in vivo* chlorophyll fluorescence: practical issues. Photosyn. Res. 122, 121–158. 10.1007/s11120-014-0024-6 25119687 PMC4210649

[B26] Katicheva L. Sukhov V. Akinchits E. Vodeneev V. (2014). Ionic nature of burn-induced variation potential in wheat leaves. Plant Cell Physiol. 55, 1511–1519. 10.1093/pcp/pcu082 24928219

[B27] Katicheva L. Sukhov V. Bushueva A. Vodeneev V. (2015). Evaluation of the open time of calcium channels at variation potential generation in wheat leaf cells. Plant Signal. Behav. 10:e993231. 10.4161/15592324.2014.993231 25738225 PMC4622019

[B28] Kenderešová L. Stanová A. Pavlovkin J. Durišová E. Nadubinská M. Ciamporová M.. (2012). Early Zn^2+^-induced effects on membrane potential account for primary heavy metal susceptibility in tolerant and sensitive *Arabidopsis* species. Ann. Bot. 110, 445–459. 10.1093/aob/mcs111 22645116 PMC3394654

[B29] Kiep V. Vadassery J. Lattke J. Maaß J. P. Boland W. Peiter E. (2015). Systemic cytosolic Ca^2+^ elevation is activated upon wounding and herbivory in *Arabidopsis*. New Phytol. 207, 996–1004. 10.1111/nph.13493 25996806

[B30] Klughammer C. Schreiber U. (2008). Saturation pulse method for assessment of energy conversion in PS I. PAM Appl. Notes 1, 11–14.

[B31] Krol E. Dziubinska H. Stolarz M. Trebacz K. (2006). Effects of ion channel inhibitors on cold- and electrically-induced action potentials in *Dionaea muscipula*. Biol. Plantarum. 50, 411–416. 10.1007/s10535-006-0058-5

[B32] Krol E. Dziubinska H. Trebacz K. (2003). Low-temperature induced transmembrane potential changes in the liverwort *Conocephalum conicum*. Plant Cell Physiol. 44, 527–533. 10.1093/pcp/pcg070 12773639

[B33] Krol E. Dziubinska H. Trebacz K. (2004). Low-temperature-induced transmembrane potential changes in mesophyll cells of *Arabidopsis thaliana, Helianthus annuus* and *Vicia faba*. Physiol. Plant. 120, 265–270. 10.1111/j.0031-9317.2004.0244.x 15032861

[B34] Krol E. Trebacz K. (1999). Calcium-dependent voltage transients evoked by illumination in the liverwort *Conocephalum conicum*. Plant Cell Physiol. 40, 17–24. 10.1093/oxfordjournals.pcp.a029470

[B35] Krupenina N. A. Bulychev A. A. (2007). Action potential in a plant cell lowers the light requirement for non-photochemical energy-dependent quenching of chlorophyll fluorescence. Biochim. Biophys. Acta 1767, 781–788. 10.1016/j.bbabio.2007.01.004 17300741

[B36] Malone M. (1994). Wound-induced hydraulic signals and stimulus transmission in *Mimosa pudica* L. New Phytol. 128, 49–56. 10.1111/j.1469-8137.1994.tb03985.x 33874540

[B37] Mancuso S. (1999). Hydraulic and electrical transmission of wound-induced signals in *Vitis vinifera*. Aust. J. Plant Physiol. 26, 55–61. 10.1071/PP98098

[B38] Maxwell K. Johnson G. N. (2000). Chlorophyll fluorescence–a practical guide. J. Exp. Bot. 51, 659–668. 10.1093/jexbot/51.345.659 10938857

[B39] Miedema H. Prins H. B. (1993). Simulation of the light-induced oscillations of the membrane potential in *Potamogeton* leaf cells. J. Membr. Biol. 133, 107–117. 10.1007/BF00233792 8515430

[B40] Mousavi S. A. R. Chauvin A. Pascaud F. Kellenberger S. Farmer E. E. (2013). GLUTAMATE RECEPTOR-LIKE genes mediate leaf-to-leaf wound signaling. Nature 500, 422–426. 10.1038/nature12478 23969459

[B41] Müller P. Li X.-P. Niyogi K. K. (2001). Non-photochemical quenching. A response to excess light energy. Plant Physiol. 125, 1558–1566. 10.1104/pp.125.4.1558 11299337 PMC1539381

[B42] Opritov V. A. Lobov S. A. Pyatygin S. S. Mysyagin S. A. (2005). Analysis of possible involvement of local bioelectric responses in chilling perception by higher plants exemplified by *Cucurbita pepo* Russ. J. Plant Physiol. 52, 801–808. 10.1007/s11183-005-0118-2

[B43] Opritov V. A. Pyatygin S. S. Krauz V. O. (1993). Role of electrical activity in cooling-induced development of adaptation syndrome in higher plant cells. Russ. J. Plant Physiol. 40, 537–542.

[B44] Opritov V. A. Pyatygin S. S. Krauz V. O. Khudyakov V. A. Abramova N. N. (1994). Activation of the electrogenic plasmalemma H^+^-pump in the adaptation of higher plants to moderate low-temperature stress. *Russ. J*. Plant Physiol. 41, 428–432.

[B45] Pavlovic A. Slováková L. Pandolfi C. Mancuso S. (2011). On the mechanism underlying photosynthetic limitation upon trigger hair irritation in the carnivorous plant Venus flytrap (*Dionaea muscipula Ellis*). J. Exp. Bot. 62, 1991–2000. 10.1093/jxb/erq404 21289078 PMC3060689

[B46] Pikulenko M. M. Bulychev A. A. (2005). Light-triggered action potentials and changes in quantum efficiency of photosystem II in *Anthoceros* cells. Russ. J. Plant Physiol. 52, 584–590. 10.1007/s11183-005-0087-5

[B47] Pyatygin S. S. (2004). Role of plasma membrane in cold action perception in plant cells. Biol. Membr. (Moscow) 21, 442–449.

[B48] Pyatygin S. S. Opritov V. A. Abramova N. N. Vodeneev V. A. (1999). Primary bioelectric response of higher plant cells to the combined action of stress factors. Russ. J. Plant Physiol. 46, 530–536.

[B49] Pyatygin S. S. Opritov V. A. Khudyakov V. A. (1992). Subthreshold changes in excitable membranes of *Cucurbita pepo* L. stem cells during cooling-induced action-potential generation. Planta 186, 161–165. 10.1007/BF00196244 24186654

[B50] Pyatygin S. S. Opritov V. A. Krauz V. O. Polovinkin A. V. (1996). Increase in cold resistance of electrogenesis as a basis for adaptive repolarization in higher plant cells during chilling. Russ. J. Plant Physiol. 43, 223–227.

[B51] Retivin V. G. Opritov V. A. Fedulina S. B. (1997). Generation of action potential induces preadaptation of *Cucurbita pepo* L. stem tissues to freezing injury. Russ. J. Plant Physiol. 44, 432–442.

[B52] Retivin V. G. Opritov V. A. Lobov S. A. Tarakanov S. A. Khudyakov V. A. (1999). Changes in the resistance of photosynthesizing cotyledon cells of pumpkin seedlings to cooling and heating, as induced by the stimulation of the root system with KCl solution. Russ. J. Plant Physiol. 46, 689–696.

[B53] Roach T. Krieger-Liszkay A. (2014). Regulation of photosynthetic electron transport and photoinhibition. Curr. Protein Pept. Sci. 15, 351–362. 10.2174/1389203715666140327105143 24678670 PMC4030316

[B54] Saidi Y. Finka A. Muriset M. Bromberg Z. Weiss Y. G. Maathuis F. J.. (2009). The heat shock response in moss plants is regulated by specific calcium-permeable channels in the plasma membrane. Plant Cell 21, 2829–2843. 10.1105/tpc.108.065318 19773386 PMC2768932

[B55] Shepherd V. A. Beilby M. J. Al Khazaaly S. A. Shimmen T. (2008). Mechano-perception in Chara cells: the influence of salinity and calcium on touch-activated receptor potentials, action potentials and ion transport. Plant Cell Environ. 31, 1575–1591. 10.1111/j.1365-3040.2008.01866.x 18684243

[B56] Sherstneva O. N. Surova L. M. Vodeneev V. A. Plotnikova Y. I. Bushueva A. V. Sukhov V. S. (2016a). The role of the intra- and extracellular protons in the photosynthetic response induced by the variation potential in pea seedlings. Biochem. (Moscow) Suppl. Ser. A 10, 60–67. 10.1134/S1990747815050116

[B57] Sherstneva O. N. Vodeneev V. A. Katicheva L. A. Surova L. M. Sukhov V. S. (2015). Participation of intracellular and extracellular pH changes in photosynthetic response development induced by variation potential in pumpkin seedlings. Biochemistry (Moscow) 80, 776–784. 10.1134/S0006297915060139 26531023

[B58] Sherstneva O. N. Vodeneev V. A. Surova L. M. Novikova E. M. Sukhov V. S. (2016b). Application of a mathematical model of variation potential for analysis of its influence on photosynthesis in higher plants. Biochem. Moscow Suppl. Ser. A 10, 269–277. 10.1134/S1990747816030089

[B59] Shimmen T. (1997). Studies on mechano-perception in characeae: effects of external Ca^2+^ and Cl^−^. Plant Cell Physiol. 38, 691–697. 10.1093/oxfordjournals.pcp.a029222 14634159

[B60] Simons P. J. (1981). The role of electricity in plant movements. New Phytol. 87, 11–37. 10.1111/j.1469-8137.1981.tb01687.x

[B61] Stahlberg R. Cleland R. E. van Volkenburgh E. (2006). Slow wave potentials – a propagating electrical signal unique to higher plants, in Communication in Plants. Neuronal Aspects of Plant Life, eds Baluška F. Mancuso S. Volkmann D. (Berlin; Heidelberg; New York, NY: Springer-Verlag), 291–308.

[B62] Stanković B. Davies E. (1996). Both action potentials and variation potentials induce proteinase inhibitor gene expression in tomato. FEBS Lett. 390, 275–279. 10.1016/0014-5793(96)00672-2 8706876

[B63] Sukhov V. (2016). Electrical signals as mechanism of photosynthesis regulation in plants. Photosyn. Res. 130, 373–387. 10.1007/s11120-016-0270-x 27154573

[B64] Sukhov V. Akinchits E. Katicheva L. Vodeneev V. (2013). Simulation of variation potential in higher plant cells. J. Membrane Biol. 246, 287–296. 10.1007/s00232-013-9529-8 23417063

[B65] Sukhov V. Orlova L. Mysyagin S. Sinitsina J. Vodeneev V. (2012). Analysis of the photosynthetic response induced by variation potential in geranium. Planta 235, 703–712. 10.1007/s00425-011-1529-2 22020752

[B66] Sukhov V. Sherstneva O. Surova L. Katicheva L. Vodeneev V. (2014a). Proton cellular influx as a probable mechanism of variation potential influence on photosynthesis in pea. Plant Cell Environ. 37, 2532–2541. 10.1111/pce.12321 24635649

[B67] Sukhov V. Surova L. Morozova E. Sherstneva O. Vodeneev V. (2016). Changes in H^+^-ATP synthase activity, proton electrochemical gradient, and pH in pea chloroplast can be connected with variation potential. Front. Plant Sci. 7:1092. 10.3389/fpls.2016.01092 27499760 PMC4956672

[B68] Sukhov V. Surova L. Sherstneva O. Katicheva L. Vodeneev V. (2015a). Variation potential influence on photosynthetic cyclic electron flow in pea. Front. Plant Sci. 5:766. 10.3389/fpls.2014.00766 25610447 PMC4285888

[B69] Sukhov V. Surova L. Sherstneva O. Bushueva A. Vodeneev V. (2015b). Variation potential induces decreased PSI damage and increased PSII damage under high external temperatures in pea. Funct. Plant Biol. 42, 727–736. 10.1071/FP15052 32480716

[B70] Sukhov V. Surova L. Sherstneva O. Vodeneev V. (2014b). Influence of variation potential on resistance of the photosynthetic machinery to heating in pea. Physiol. Plant. 152, 773–783. 10.1111/ppl.12208 24730552

[B71] Sukhov V. Vodeneev V. (2009). A mathematical model of action potential in cells of vascular plants. J. Membrane Biol. 232, 59–67. 10.1007/s00232-009-9218-9 19921324

[B72] Sukhova E. Akinchits E. Sukhov V. (2017). Mathematical models of electrical activity in plants. J. Membrane Biol. 250, 407–423. 10.1007/s00232-017-9969-7 28711950

[B73] Surova L. Sherstneva O. Vodeneev V. Katicheva L. Semina M. Sukhov V. (2016a). Variation potential-induced photosynthetic and respiratory changes increase ATP content in pea leaves. J. Plant Physiol. 202, 57–64. 10.1016/j.jplph.2016.05.024 27450494

[B74] Surova L. Sherstneva O. Vodeneev V. Sukhov V. (2016b). Variation potential propagation decreases heat-related damage of pea photosystem I by 2 different pathways. Plant Sign. Behav. 11:e1145334. 10.1080/15592324.2016.1145334 26853242 PMC4883963

[B75] Trebacz K. Dziubinska H. Krol E. (2006). Electrical signals in long-distance communication in plants, in Communication in *Plants* Neuronal *Aspects* of *Plant Life*, eds Baluška F. Mancuso S. Volkmann D. (Berlin; Heidelberg: Springer-Verlag), 277–290.

[B76] Trebacz K. Sievers A. (1998). Action potentials evoked by light in traps of *Dionaea muscipula* Ellis. Plant Cell Physiol. 39, 369–372. 10.1093/oxfordjournals.pcp.a029379

[B77] Trebacz K. Simonis W. Schönknecht G. (1997). Effects of anion channel inhibitors on light-induced potential changes in the liverwort *Conocephalum conicum*. Plant Cell Physiol. 38, 550–557. 10.1093/oxfordjournals.pcp.a029204

[B78] Vodeneev V. A. Akinchits E. K. Orlova L. A. Sukhov V. S. (2011). The role of Ca^2+^, H^+^, and Cl^−^ ions in generation of variation potential in pumpkin plants. Russ. J. Plant Physiol. 58, 974–981. 10.1134/S1021443711050256

[B79] Vodeneev V. A. Katicheva L. A. Sukhov V. S. (2016). Electrical signals in higher plants: mechanisms of generation and propagation. Biophysics 61, 505–512. 10.1134/S0006350916030209

[B80] Vodeneev V. Akinchits E. Sukhov V. (2015). Variation potential in higher plants: mechanisms of generation and propagation. Plant Signal. Behav. 10:e1057365. 10.1080/15592324.2015.1057365 26313506 PMC4883923

[B81] Vodeneev V. Mudrilov M. Akinchits E. Balalaeva I. Sukhov V. (2017). Parameters of electrical signals and photosynthetic responses induced by them in pea seedlings depend on the nature of stimulus. Funct. Plant Biol. 10.1071/FP16342 32291030

[B82] Volkov A. G. Ranatunga D. R. A. (2006). Plants as environmental biosensors. Plant Signal Behav. 1, 105–115. 10.4161/psb.1.3.3000 19521490 PMC2635006

[B83] Von Caemmerer S. Farquhar G. D. (1981). Some relationships between the biochemistry of photosynthesis and the gas exchange of leaves. Planta 153, 376–387. 10.1007/BF00384257 24276943

